# Cyrus A. Raji, MD, PhD, is the recipient of the 2025 Mark A. Smith Alzheimer Award

**DOI:** 10.1177/13872877251361092

**Published:** 2025-07-28


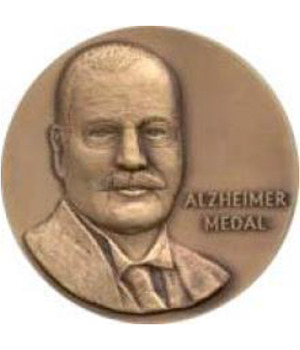
.

The Journal of Alzheimer's Disease (JAD) is pleased to announce that **Cyrus A. Raji, MD, PhD,** is the recipient of the 2025 Mark A. Smith Alzheimer Award. The award is presented by the journal in recognition of Cyrus Raji and colleagues’ groundbreaking article “Exercise-Related Physical Activity Relates to Brain Volumes in 10,125 Individuals”.^1^ The awardee is selected by the journal's Editorial Board from the 2024 year's volume (800 papers), and is presented with the Alzheimer Medal, a 3” bronze medal with the likeness of Alois Alzheimer, and a cash prize of $7500. The 2025 award is proudly sponsored by IOS Press/Sage Publications.


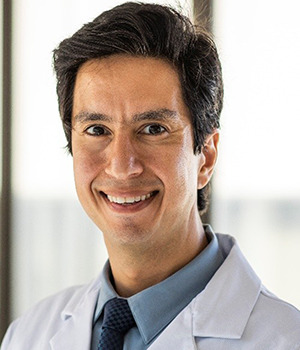
.

Cyrus A. Raji, MD, PhD, is a tenured associate professor of radiology and neurology as well as a principal investigator in the Neuroimaging Labs Research Center at Mallinckrodt Institute of Radiology (MIR), the academic radiology department of Washington University School of Medicine in St Louis. In addition, he directs the Raji Brain Health Imaging Lab. Additionally, he serves as director of Neuromagnetic Resonance Imaging at Barnes-Jewish Hospital and as associate director of the diagnostic radiology residency research track.

Dr Raji obtained his MD and PhD degrees from the University of Pittsburgh School of Medicine where he was part of pioneering work on the application of amyloid imaging in Alzheimer's disease. He then did his internship at UPMC-Mercy Hospital before doing a four-year diagnostic radiology residency at UCLA Medical Center where his research won the Radiological Society of North America Roentgen Award for Outstanding Radiological Research. He then completed a two-year Neuroradiology fellowship at UCSF that included an NIH funded research year on diffusion MRI in traumatic brain injury—a known risk factor for dementia.

Dr Raji is board-certified in diagnostic radiology and neuroradiology with research interests focusing on modifiable risk factors for dementia such as obesity, physical activity, as well as traumatic brain injury in addition to the role of advanced neuroimaging in quantitatively tracking related brain changes. He has published over 100 peer-reviewed articles on these topics and has been cited over 6600 times. His NIH funded research has been featured in multiple media outlets including CNN, Fox News, the New York Times and USA Today. Dr Raji is also recognized as a 2024 Castle Connelly Top Doctor in Neuroradiology, MRI, and traumatic brain injury.

## Importance of published article

This paper combined physical activity data from a large cohort of over 10,000 individuals with quantitative MRI data to demonstrate that moderate to vigorous physical activity—as little as 25 min per week—is related to larger brain volumes including in areas that are vulnerable to Alzheimer's pathology: the hippocampus, posterior cingulate gyrus and precuneus. This work was cited in the Lancet 2024 Commission on Dementia Prevention (reference #181) as supporting evidence for physical activity as a way to reduce the risk for dementia, including Alzheimer's. This work thus illustrates the key importance of imaging brain health. The concept of brain heath posits that the absence of disease must also be coupled with the presence of optimal structural, physiological, and molecular integrity of the human brain. Brain health imaging utilizes multimodal neuroimaging techniques to identify such changes. These include various MRI parameters that can identify brain atrophy, neuroinflammation, and lower cerebral perfusion as well as molecular imaging tracers on positron emission tomography to track Alzheimer's disease neuropathology. Understanding brain health also requires integrating insights from the periphery that in case of obesity entails quantitative measurements of visceral and subcutaneous fat as well as liver fat and fatty replacement of the musculature in the setting of sarcopenia. Advancing these key domains of knowledge will allow for improved preventive approaches for Alzheimer's disease that target disorders of critical public health relevance such as obesity while highlighting as our paper did, strategies such as physical activity that can also lower risk for Alzheimer's disease.
